# Gap junctions and hemichannels in cancer: A “Wired versus Broadcast” framework for tumor microenvironment routing of cGAMP–STING signals and therapy response

**DOI:** 10.1016/j.tranon.2026.103002

**Published:** 2026-09-15

**Authors:** Soroush Akbari-Ardabili, Safiyeh Aghazadeh, Ali Shalizar-Jalali, Faezeh Hosseinzadeh

**Affiliations:** a Cellular and Molecular Research Centre, Qom University of Medical Sciences, Qom, Iran; b Department of Basic Sciences, Faculty of Veterinary Medicine, Urmia University, Urmia, Iran; c Department of Tissue Engineering and Applied Cell Sciences, Faculty of Medicine, Qom University of Medical Sciences, Qom, Iran

**Keywords:** Gap junction, Connexin 43, Pannexin 1, cGAMP–STING pathway, ENPP1

## Abstract

Intercellular communication in the tumor microenvironment (TME) dictates cancer progression, immune evasion, and therapy response. Gap junctions (GJs, primarily connexins), connexin hemichannels, and pannexin channels have been widely reviewed, but a unified conceptual model that contrasts protected “Wired” versus vulnerable “Broadcast” routing of cGAMP and ATP in the TME, and links this routing to therapy response, has been lacking. We propose a “Wired vs Broadcast” framework to define the routing of TME danger signals. “Wired” signaling, via connexin 43 (Cx43) gap junction intercellular communication, mediates contact-dependent, protected transfer of the immunotransmitter 2´3´-cGAMP from tumor cells to recipient cells, activating the STING pathway. “Broadcast” signaling, via PANX1 channels and related extracellular release routes, releases ATP and extracellular cGAMP into the TME, where they are intercepted and neutralized by ectoenzymes such as ENPP1 and CD39/CD73. This framework highlights context-dependent, recipient-cell–dependent outcomes of “Wired” signaling: a tumor-to–antigen-presenting cell (APC) “Wired” circuit can drive anti-tumor immunity and support immune checkpoint blockade (ICB) responsiveness, whereas a tumor-to-astrocyte “Wired” circuit can promote brain metastasis and chemoresistance. The relative contributions of “Wired” and “Broadcast” routing, shaped by cellular proximity, scaffolding proteins, transport and uptake mechanisms, and ectoenzyme expression, may influence therapeutic response in a context-dependent manner. This review synthesizes current evidence into an integrative and testable “Wired versus Broadcast” framework and discusses biomarker and combination-therapy hypotheses that can be evaluated in forthcoming spatial-omics and clinical studies.

## Highlights

• Wired vs Broadcast framework maps nucleotide routing in the cancer TME.
• Cx43 gap junctions can route cGAMP to APCs and support STING–ICB responses.
• ENPP1 and CD39/CD73 shape vulnerable extracellular cGAMP/ATP signaling.
• Cx43 wiring diverges between tumor–APC immunity and tumor–astrocyte metastasis.
• Biomarker and RT/ICB combinations are proposed as testable strategies.

## Graphical abstract

Context-dependent outcomes of Cx43-wired cGAMP transfer: Tumor→APC anti-tumor signaling versus tumor→astrocyte pro-tumor signaling.

**Figure:**
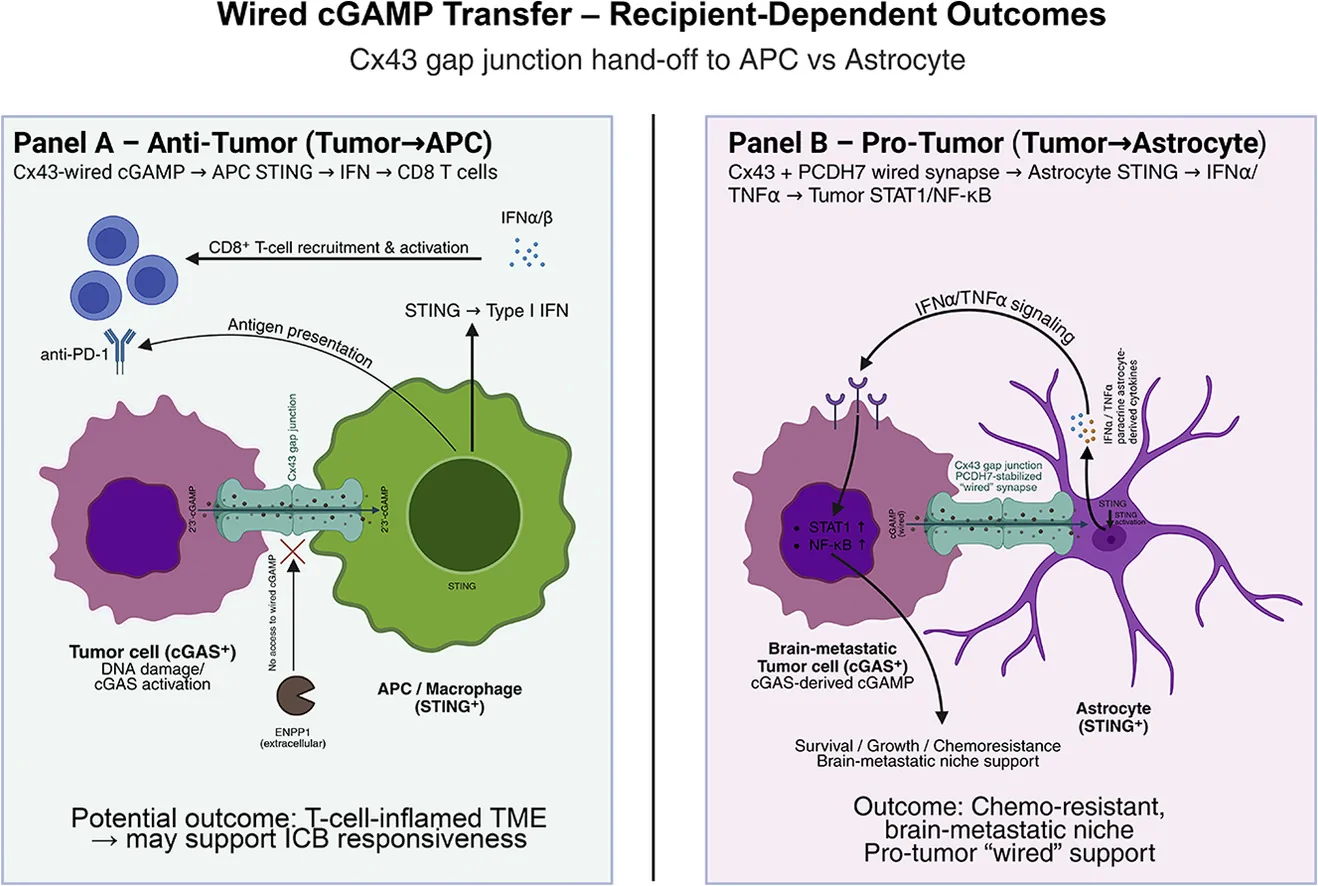

## Introduction

Gap junctions, connexins, pannexins, cGAMP–STING signaling, and ectonucleotide checkpoints have each been reviewed extensively as separate components of cancer biology and tumor immunity [[1], [2], [3],[22], [23], [24], [25], [26], [27], [28], [29],^50^,^52^,^53^]. However, these literatures have rarely been integrated around the topological problem that determines whether a nucleotide danger signal is protected during cell-to-cell transfer or exposed to extracellular degradation and conversion in the tumor microenvironment. This distinction is particularly important for cGAMP and ATP, whose biological effects depend not only on their production but also on their route of delivery, recipient-cell identity, and exposure to ectoenzymes such as ENPP1 and CD39/CD73 [[22], [23], [24], [25], [26], [27], [28],^50^].

**Fig. 1:**
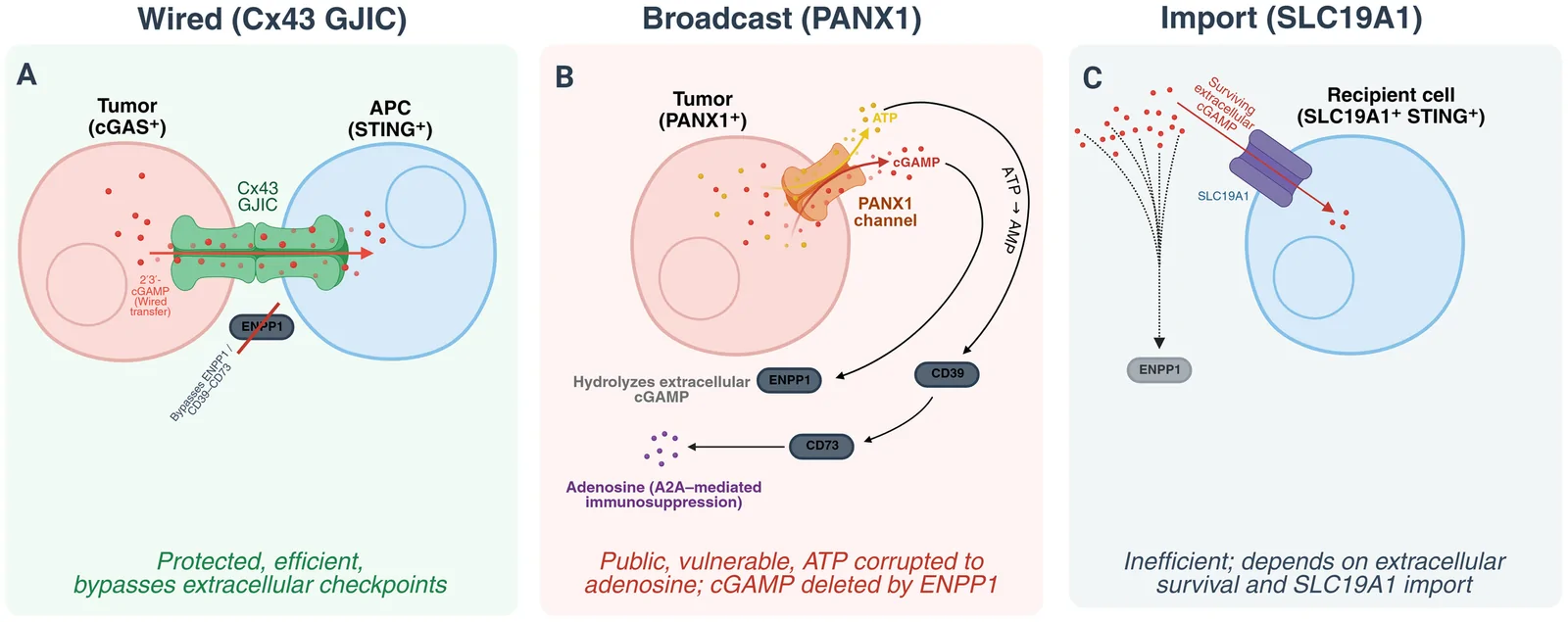
Mechanism comparison: Wired gap junction intercellular communication (GJIC) vs Broadcast (PANX1) vs Importers. The "Wired vs. Broadcast" framework contrasts protected versus vulnerable signaling routes. Panel A shows the “Wired” route, in which Cx43 GJIC creates a private, cytosol-to-cytosol tunnel. 2´3´-cGAMP is transferred directly from donor tumor cells to recipient cells without extracellular exposure, thereby bypassing extracellular checkpoints. Panel B shows the “Broadcast” route, in which PANX1 channels and related extracellular-release routes expose ATP and cGAMP to the extracellular tumor microenvironment. This “Broadcast” signal is vulnerable to extracellular checkpoint activity: ATP is converted by CD39/CD73 into immunosuppressive adenosine, whereas cGAMP is hydrolyzed by the ectoenzyme ENPP1, reducing its availability for paracrine STING activation. Panel C shows extracellular cGAMP uptake through representative importer mechanisms; SLC19A1 is shown as one example, and additional context-dependent import routes, including LRRC8/VRAC channels and P2X7-linked mechanisms, are discussed in the text. Among currently characterized routes, the “Wired” Cx43 pathway is the only well-defined mechanism that delivers cGAMP directly from one cytosol to another while bypassing extracellular checkpoints.

**Fig. 2:**
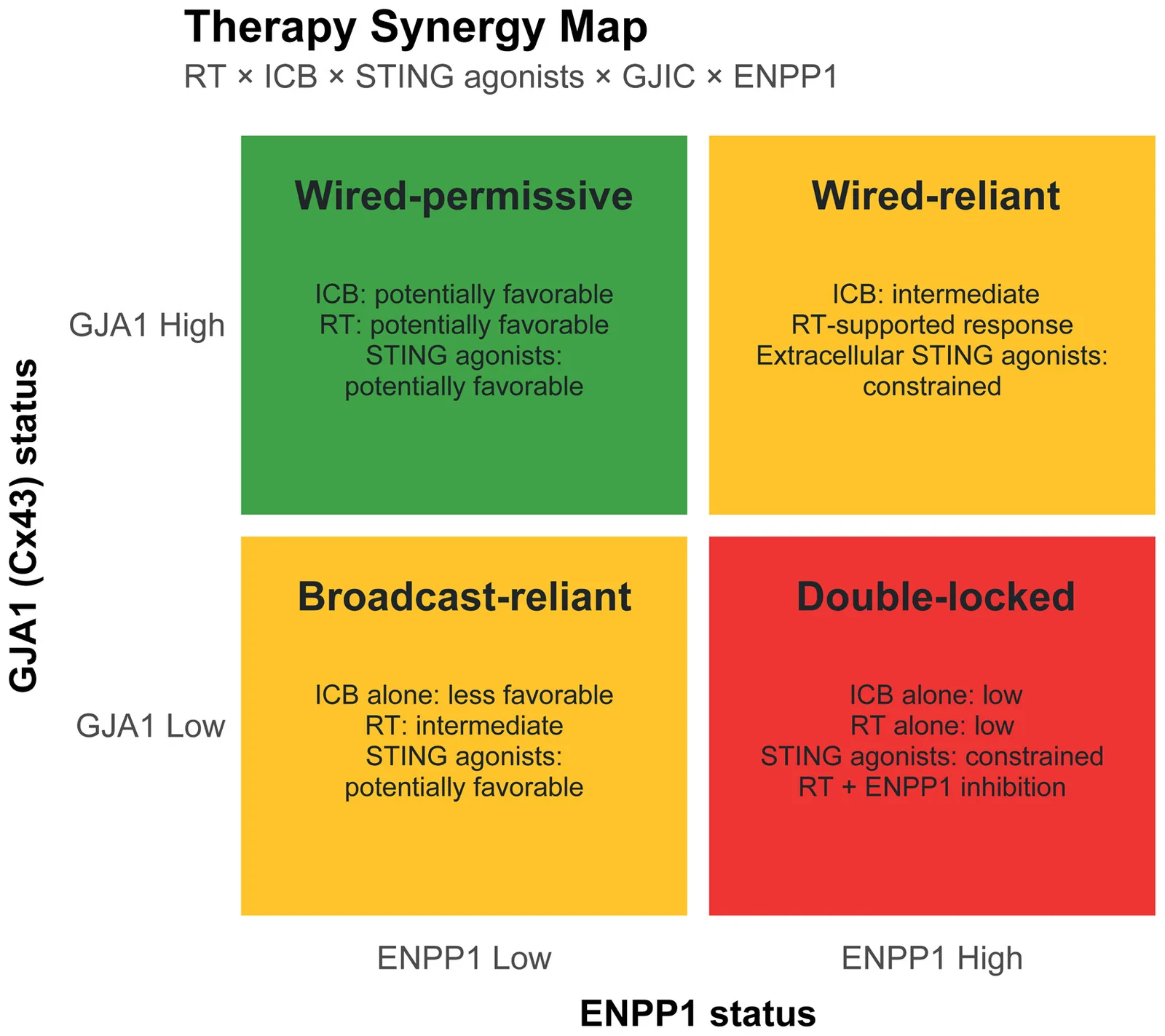
Therapy synergy map: radiotherapy (RT), immune checkpoint blockade (ICB), and STING agonists × “Wired” (GJA1) × “Broadcast” (ENPP1) status. The “Wired vs Broadcast” framework provides a working model for therapeutic behavior according to tumor “Wired” (GJA1) and “Broadcast” (ENPP1) status. GJA1-high / ENPP1-low (“Wired-permissive”): both “Wired” Cx43 and “Broadcast” cGAMP routes are potentially available; these tumors may be more likely to respond to ICB, RT, and STING-directed strategies. GJA1-high / ENPP1-high (“Wired-reliant”): the “Broadcast” cGAMP route is constrained by ENPP1, so RT-induced cGAMP signaling may rely more heavily on the protected Cx43 route; these tumors may benefit from RT + ICB, whereas extracellularly delivered STING agonists may be less effective. GJA1-low / ENPP1-low (“Broadcast-reliant”): the “Wired” route is impaired, but the “Broadcast” route remains relatively permissive; RT or STING agonists may enhance response if extracellular cGAMP survives and is imported by recipient cells. GJA1-low / ENPP1-high (“Double-locked”): both “Wired” transfer and “Broadcast” cGAMP signaling are constrained; these tumors may represent rational candidates for combinations that relieve ENPP1-mediated extracellular cGAMP loss, such as RT + ENPP1 inhibition. This matrix is hypothesis-generating and is not intended for clinical stratification or treatment selection without prospective validation.

**Fig. 3:**
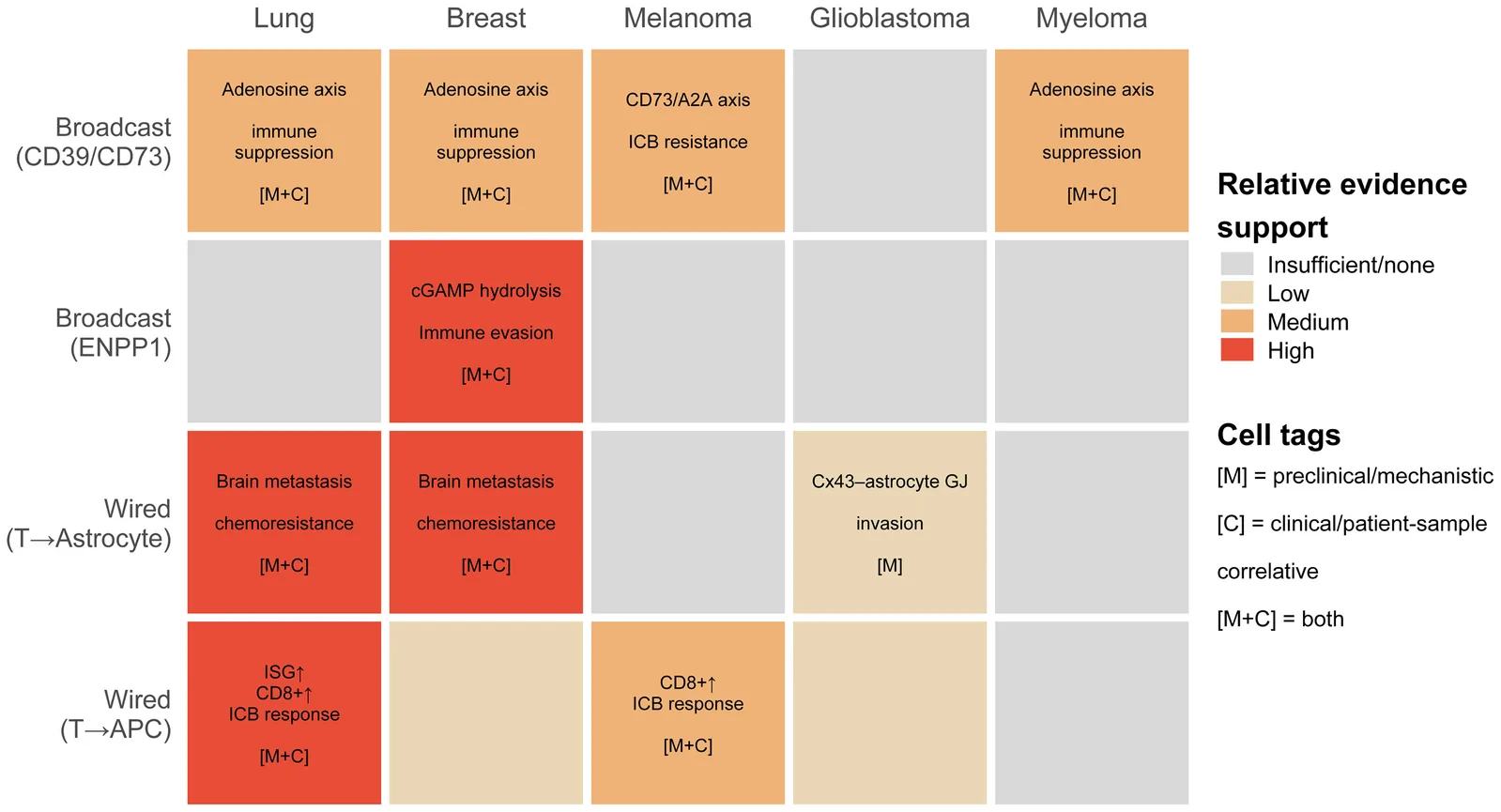
Evidence map: cancer types, signaling circuits, immune readouts, and evidence type. Evidence map for the “Wired vs. Broadcast” framework across selected cancer contexts. Color intensity indicates relative evidence support (insufficient/none, low, medium, high), whereas cell labels indicate the dominant biological readout associated with each circuit. Evidence-type tags distinguish preclinical/mechanistic model evidence [M], clinical or patient-sample correlative evidence [C], and support from both evidence layers [M+C]. The anti-tumor tumor→APC “Wired” circuit (Cx43→APC→STING) is supported most directly in lung cancer, with additional melanoma-related support, where GJA1/Cx43 activity has been linked to interferon-stimulated gene (ISG) programs, CD8+ T-cell infiltration, and immune checkpoint blockade (ICB) responsiveness. The pro-tumor tumor→astrocyte “Wired” circuit (Cx43/PCDH7→astrocyte→STING) is most strongly supported in brain-metastasis models from breast and lung cancer, with clinical correlative links to brain metastatic tropism and therapy resistance. The “Broadcast” checkpoint layer is represented separately for CD39/CD73-mediated ATP-to-adenosine conversion and ENPP1-mediated extracellular cGAMP hydrolysis, because these mechanisms generate distinct but convergent routes toward immune suppression or reduced STING signaling. The distribution of colored cells reflects the availability of context-specific evidence rather than the presumed biological absence or presence of each pathway. Blank or pale cells indicate contexts where evidence is absent, indirect, or not yet sufficiently developed. This map is intended as a qualitative synthesis rather than a quantitative meta-analysis.

The aim of this review is therefore to synthesize current evidence into a “Wired versus Broadcast” framework for channel-dependent routing of nucleotide danger signals in cancer. We use “Wired” to describe protected, contact-dependent cytosol-to-cytosol transfer through Cx43 gap junctional intercellular communication, and “Broadcast” to describe extracellular release through connexin hemichannels, PANX1 channels, or other export routes that expose cGAMP and ATP to the tumor microenvironment. These terms denote operational routing modes rather than mutually exclusive biological states, because gap-junctional transfer may coexist with extracellular release, vesicular transport, and importer-dependent uptake within the same tumor, with their relative contributions varying across cell types and spatial niches. We focus on Cx43/GJA1 because it is the most extensively studied connexin in cancer biology and because the most direct evidence for intercellular cGAMP transfer in the tumor microenvironment currently involves Cx43-mediated coupling. Other connexins may also contribute to junctional signaling in selected contexts, but their role in cGAMP routing remains less directly characterized. This framework should be interpreted as an integrative and testable conceptual model, not as a validated clinical classification. Its value lies in organizing existing evidence into experimentally tractable hypotheses about tumor–APC coupling, tumor–astrocyte coupling, extracellular checkpoint activity, and therapy-response stratification. Because the supporting evidence is uneven across cancer types, we treat these circuits as tumor-context-dependent modules rather than universal mechanisms that apply uniformly to all malignancies. The overall routing logic is summarized schematically in Fig. 1, the principal circuit types are summarized in Table 1, the therapeutic working matrix is shown in Fig. 2, and the tumor-context evidence map is shown in Fig. 3.

**Table 1:** Circuit inventory: Dyad | Channel/isoform | Permeant(s) | Recipient readout | Net effect | Evidence type | DOI/PMID.

Circuit Type	Dyad (Donor → Recipient)	Channel/Scaffold	Permeant(s) / Substrate	Recipient Readout	Net Effect on Tumor	Key Evidence
**Wired**	Tumor → APC (Macrophage/DC)	Cx43 (*GJA1*)	2´3´-cGAMP	APC-STING → Type I IFN	**Anti-Tumor** / ICB Sensitization	PMID: 40437141 DOI: 10.1007/s12032–025–02773-7
**Wired**	Tumor → Astrocyte	Cx43 (*GJA1*) + PCDH7	2´3´-cGAMP	Astrocyte-STING → IFNα/TNFα → Tumor STAT1/NF-κB	**Pro-Tumor** / Brain Metastasis / Chemoresistance	PMID: 27225120 DOI: 10.1038/nature18268
**Broadcast**	Tumor → TME	PANX1	ATP, UTP	T-Cell A2AR (via CD39/CD73)	**Immunosuppression** (via Adenosine)	PMID: 35884429 DOI: 10.3390/cancers14143369
**Broadcast**	Tumor → TME	Export (PANX1, Vesicles)	Extracellular 2´3´-cGAMP	ENPP1 Hydrolysis	**Immune Evasion (Signal Hydrolysis)**	PMID: 33768207 DOI: 10.1038/s43018–020–0028–4
**Boundary**	TME → Host Cell	SLC19A1	Extracellular 2´3´-cGAMP	Host Cell-STING	Paracrine Immunity (if ENPP1 is low)	PMID: 31126740 DOI: 10.1016/j.molcel.2019.05.006

APC, antigen-presenting cell; cGAMP, cyclic GMP–AMP; DC, dendritic cell; ICB, immune checkpoint blockade; IFN, interferon; TME, tumor microenvironment.

## Literature search and evidence synthesis

This article is a targeted narrative review. Relevant literature available through March 2026 was identified through focused searches of PubMed/MEDLINE and Google Scholar, supplemented by citation tracking from key primary studies and recent reviews. Search concepts included combinations of terms related to connexins, Cx43/GJA1, pannexins/PANX1, gap junctional intercellular communication, hemichannels, cGAS–cGAMP–STING signaling, extracellular cGAMP, ENPP1, CD39/CD73, adenosine signaling, immune checkpoint blockade, radiotherapy, brain metastasis, astrocytes, and antigen-presenting cells. Priority was given to peer-reviewed mechanistic studies, translational cancer studies, and recent reviews directly relevant to nucleotide-signal routing in the tumor microenvironment. Evidence summarized in Fig. 3 was classified qualitatively according to relative support and evidence type, distinguishing preclinical/mechanistic model evidence from clinical or patient-sample correlative evidence. This review was not designed as a systematic review or meta-analysis.

## Biochemistry of gap junctions and hemichannels: architecture, gating, and protein–protein interactions

Intercellular communication in cancer is governed by a fundamental choice: the transfer of signals through a protected "Wired" channel or their public "Broadcast" into the tumor microenvironment (TME) [1,2]. The architecture of connexin (Cx) and pannexin (PANX) channels forms the basis of this decision [3]. In addition to these mammalian channel families, innexins are the invertebrate functional analogues of connexins and form gap junctions in organisms such as *Drosophila* and *C. elegans* [4,5]. In this review, however, we focus exclusively on connexin-based “Wired” and pannexin-based “Broadcast” signaling in human cancer. Accordingly, the framework proposed here is intentionally restricted to channel-dependent routing of nucleotide danger signals, particularly cGAMP and ATP, and does not aim to explain the full spectrum of connexin biology, including channel-independent effects on cytoskeletal organization, transcriptional regulation, or other non-junctional signaling functions [52,53,[56], [57], [58]].

A "Wired" connection, or gap junction (GJ), is formed by clusters of intercellular channels, each generated by the docking of two hexameric connexons, one contributed by each apposed cell [3]. These junctional channels facilitate the direct transfer of small hydrophilic molecules and ions, generally below approximately 1 kDa, through gap junctional intercellular communication (GJIC) [6]. In contrast, “Broadcast” signaling can occur through extracellular-release routes, including connexin hemichannels and, more prominently in the TME, structurally distinct PANX1 channels that open to the extracellular space [7]. This topological contrast, an apposed cell–cell junction versus a pore that opens directly to the TME, defines the first “Wired versus Broadcast” decision point in this framework. Strictly, the term “hemichannel” is most often used for undocked connexons, whereas pannexins form structurally distinct large-pore channels. We therefore use “PANX1 channels” for pannexin-mediated Broadcast signaling and reserve “connexin hemichannels” for undocked connexons unless otherwise specified.

The “Wired” circuit is not a static structure; it is dynamically gated by a complex “phospho-code” on the C-terminal tail of Cx43, the most widely expressed connexin [8]. This code determines “Wired” circuit stability and conductance. Activation of specific Protein Kinase C isoforms (for example PKCα and PKCδ), often by TME-derived growth factors (e.g., VEGF) or oxidative stress, leads to phosphorylation of serine 368 (pS368) [8]. This pS368 mark acts as a “close” signal, inducing rapid channel closure, altering conductance to favor smaller ions over larger permeants such as cGAMP, and triggering clathrin-dependent internalization [9]. Similarly, activation of MAPK pathways such as ERK1/2 phosphorylates serines S279 and S282 [10]. These sites serve as a “turnover” signal, increasing the binding affinity for the E3-ligase NEDD4 and tagging the connexon for ubiquitination and degradation [11,12]. Experimentally, PKC activity can be enhanced with phorbol esters or inhibited with small molecules such as GF109203X, whereas ERK signaling can be blocked with MEK inhibitors such as U0126; together, these reagents provide concrete tools to dissect the Cx43 phospho-code in vitro and in vivo [13,14].

From a translational perspective, this phospho-code also implies that total GJA1/Cx43 abundance alone may be an incomplete biomarker of “Wired” competence. A tumor may express substantial total Cx43 but still remain functionally “unwired” if Cx43 is retained in non-junctional cytoplasmic pools or if a large fraction of the junctional channel pool is phosphorylated at closure- or turnover-associated sites such as pS368 or S279/S282 [[8], [9], [10],^39^]. Accordingly, phospho-specific Cx43 readouts should be considered as functional biomarker candidates rather than simple expression markers. In future spatial pathology studies, total Cx43, phospho-Cx43 states, and tumor–APC proximity could be assessed together to distinguish merely Cx43-positive tumors from tumors with a potentially permissive “Wired” signaling state.

The "Wired" infrastructure also relies on specific protein-protein interactions (PPIs) for assembly and removal. The tight-junction scaffolding protein Zonula Occludens-1 (ZO-1) is essential for the transition of individual connexons into stable GJ plaques [15]. ZO-1 binds the C-terminus of Cx43 via its second PDZ domain, anchoring the channel complex [16]. Disruption of this Cx43–ZO-1 interaction destabilizes the "Wired" synapse and shifts the balance from gap junctional coupling toward connexin hemichannel activity [15]. Conversely, channel removal is regulated by endocytosis, including a mechanism linked to caveolin-1 (Cav-1) and lipid rafts, which facilitates the rapid internalization of GJs to modulate "Wired" circuit availability [9,17].

The "Broadcast" layer, dominated here by PANX1, is also tightly gated [3,7]. We focus on PANX1 because it is the best-characterized pannexin in tumor-associated ATP release and extracellular nucleotide signaling; PANX2 and PANX3 may have context- and cell-type-dependent roles, but comparatively less direct evidence links them to the cGAMP/ATP Broadcast circuits considered here. PANX1 channels are primarily known for the robust efflux of ATP [7,18]. This channel is activated by stimuli such as caspase-mediated C-terminal cleavage during apoptosis or by functional coupling to the purinergic receptor P2X7 [19]. Furthermore, some metastatic cells, particularly in breast cancer, are enriched for a truncated, constitutively active form (Panx11–89) that supports a sustained “Broadcast” of ATP, promoting survival and metastasis [20,21]. This provides a mechanism for tumors to maintain a high-ATP "Broadcast" state [20,21].

Once a signal is "Broadcast," it enters a high-interference environment defined by extracellular checkpoints [22,23]. ATP "Broadcast" by PANX1 is immediately intercepted by the ectonucleotidase cascade of CD39 (which converts ATP to AMP) and CD73 (which converts AMP to adenosine) [22,24]. In many tumors, this “Broadcast” ATP is rapidly converted into adenosine by CD39/CD73, shifting a pro-inflammatory DAMP into a potently immunosuppressive metabolite that suppresses T-cell function via A2A receptors [25,26]. Simultaneously, "Broadcast" 2´3´-cGAMP is targeted by its primary extracellular hydrolase, ENPP1 [27]. ENPP1 effectively hydrolyzes this signal, reducing its availability for paracrine STING activation [28]. This TME biochemistry establishes the central dilemma: the "Wired" Cx43 route offers a protected, private transfer that *bypasses* these extracellular checkpoints, whereas the "Broadcast" PANX1/export route is a public, vulnerable signal that is actively neutralized or redirected [22,23,27].

## Mechanistic core: Cx43-wired cGAMP transfer and STING activation

The canonical cGAS–STING pathway is a central sensor of innate immunity [[29], [30], [31]]. Cytosolic double-stranded DNA (dsDNA), generated by genomic instability, micronuclei formation, or DNA damage, binds the enzyme cyclic-GMP-AMP synthase (cGAS, MB21D1), which synthesizes the second messenger 2´3´-cGAMP [29,32]. cGAMP, in turn, binds the endoplasmic reticulum-resident adaptor STING (*TMEM173*), inducing its translocation [33]. Activated STING recruits and activates TBK1, which phosphorylates IRF3 (driving Type I interferon, IFN, production) and IKK (activating NF-κB and inflammatory cytokines) [31,33]. While this pathway is often considered cell-autonomous, its role in tumor immunity is defined by the intercellular transfer of its key signal, cGAMP [34,35].

The "Wired" hand-off of cGAMP is the mechanistic core of this framework. cGAMP is a small (≈675 Da), polar molecule that fits comfortably within the permissive permeant window of an open Cx43 gap junction (which allows passage of molecules ≤ 1 kDa) [36]. This enables a direct, "Wired" transfer: a cGAS-positive donor cell (e.g., a tumor cell) produces cGAMP, which then diffuses through the Cx43 GJIC into the cytosol of a STING-positive recipient cell (e.g., an APC) [35,37]. This intercellular transfer, first identified as a mechanism for spreading antiviral immunity, is now understood to be a critical event in cancer immunology [37,38]. The “Wired” route is currently the only well-defined mechanism that delivers cGAMP from one cell’s cytosol directly into another’s while completely shielding it from the extracellular TME [37,38].

A successful "Wired" cGAMP transfer is not automatic; it is a contingent event requiring several biophysical and biochemical prerequisites [39,40]. First, stable cell–cell physical contact is non-negotiable [41]. Second, the cells must express sufficient surface levels of Cx43 (*GJA1*) to form GJs [39,40,42]. Third, these channels must be in a permissive, open-gated state, implying a favorable "phospho-code" (e.g., low PKC-mediated pS368 activity) [36,39]. Fourth, the "Wired" infrastructure must be assembled and stabilized, a process reliant on intact ZO-1 scaffolding [15,43,44]. The absence of any of these factors will break the "Wired" circuit [39,40].

Failure of the "Wired" circuit can occur through multiple mechanisms. Physical separation by extracellular matrix (ECM) or the presence of non-coupling stromal cells (e.g., certain fibroblasts) prevents GJ formation [45,46]. Active downregulation of *GJA1* expression is a common feature in many advanced cancers and is directly correlated with resistance to immunotherapy [40,42]. Furthermore, active signaling within the TME can rapidly dismantle existing "Wired" connections, such as through pS368-driven internalization [10,39]. Finally, the "Wired" circuit fails if a suitable recipient (e.g., an APC) is not in proximity [40].

Together, these factors shape the relative contributions of “Wired” and “Broadcast” routes in a cGAMP-producing tumor cell [34,35]. If a stable "Wired" synapse exists with an APC (Cx43-high, proximity-high), cGAMP is transferred *directly* and *safely* [35,40]. When functional “Wired” coupling is limited or absent, cGAMP signaling may rely more heavily on extracellular release mechanisms, including vesicular and other export routes [35,68]. This "Broadcast" cGAMP immediately encounters the ENPP1 checkpoint [23,47]. High ENPP1 expression in the TME, a feature correlated with immune evasion and metastasis, rapidly hydrolyzes the "Broadcast" cGAMP, attenuating the immune signal before it can activate paracrine STING in host cells [23,48,49]. The "Wired" Cx43 GJ thus functions as a "private tunnel" to bypass this ENPP1 "public checkpoint" [23,40]. This topological advantage may help explain why Cx43 expression has been linked to ICB responsiveness: in ENPP1-high or importer-limited TMEs, Cx43-mediated GJIC can provide a protected route for cGAMP transfer that is less exposed to extracellular loss than Broadcast export [40,48].

While GJs handle the direct cytosol-to-cytosol "Wired" transfer, it is important to note the boundary conditions set by "Broadcast" import [50]. Extracellular "Broadcast" cGAMP, if it survives ENPP1 hydrolysis (i.e., in an ENPP1-low TME), must still cross the plasma membrane of recipient cells to activate STING [47]. The folate transporter SLC19A1 has been identified as an important importer for cGAMP and other cyclic dinucleotides (CDNs) [51]. However, cGAMP uptake is not restricted to SLC19A1. LRRC8-containing volume-regulated anion channels (VRACs), including LRRC8A/C- or LRRC8A/E-containing channels, can mediate context-dependent CDN transport, and P2X7 receptor activity has also been linked to ATP-dependent cGAMP uptake in macrophage-rich tumor settings [50,68]. Thus, the vulnerability of the "Broadcast" route is not simply extracellular degradation by ENPP1; it also depends on which import machinery is present, active, and spatially aligned with STING-competent recipient cells.

## Contextual circuits in the tumor microenvironment: tumor→APC (anti-tumor) versus tumor→astrocyte (pro-tumor)

The "Wired vs. Broadcast" framework demonstrates that the *routing* of a signal is as important as the signal itself [52,53]. This is most evident in the "Wired" circuit, where the *Cx43 → cGAMP → STING* mechanism is biochemically identical in opposing circuits [54,55]. The net outcome, anti-tumor immunity versus pro-tumor progression, is largely dictated by the *identity of the recipient cell* coupled to the “Wired” channel [54,55]. This contextual dependency offers a mechanistic lens that helps clarify one channel-dependent aspect of the long-standing paradox of connexins acting as both tumor suppressors and promoters, particularly within the cGAMP–STING/TME-routing axis, rather than resolving all facets of connexin biology [52,54,55]. Other Cx43 functions, including isoform-specific behavior, cytoskeletal modulation, transcriptional or gene-regulatory effects, non-junctional scaffolding roles, and additional post-translational modifications, fall outside the direct scope of this routing framework except where they affect gap-junctional coupling or nucleotide transfer [52,53,[56], [57], [58]]. This section contrasts the two most prominent and functionally opposite "Wired" circuits.

The anti-tumor circuit involves a "Wired" hand-off from tumor cells to antigen-presenting cells (APCs) [55]. In models of lung cancer and melanoma, tumor cells with high Cx43 expression form functional GJs with adjacent APCs, such as macrophages and dendritic cells (DCs) [55]. Tumor-derived cGAMP, produced in response to genomic instability, is transferred via this "Wired" Cx43 synapse into the APC cytosol [35]. This activation of STING *in the APC* is the critical step [59]. APCs are specialized immune cells capable of antigen presentation, co-stimulation, and interferon-driven T-cell priming [59]. APC-STING activation drives their maturation, enhances antigen cross-presentation, and unleashes a potent Type I IFN program [59]. This, in turn, promotes the recruitment, activation, and cytotoxic function of CD8+ T cells, creating an inflamed, T-cell-rich TME [59].

This T→APC "Wired" circuit is emerging as a critical prerequisite for sensitivity to immune checkpoint blockade (ICB) [40,60]. The anti-tumor T-cell response driven by the T → APC → STING → IFN axis is the very process that ICB (e.g., anti-PD-1) aims to "release the brakes" on [40,60]. Preclinical evidence supports this model: silencing GJA1 (Cx43) in lung cancer cells prevents cGAMP transfer to macrophages [40,55]. This loss of "Wired" signaling inhibits STING activation in the macrophages, causing them to polarize toward a pro-tumor M2 (TAM) phenotype [61]. The resulting TME becomes "cold," with reduced CD8+ T-cell activation and reduced sensitivity to anti-PD-1 therapy [60]. In this context, Cx43 expression may reflect, and in part enable, the pro-immunogenic "Wired" synapse that supports ICB efficacy [40,60].

In stark contrast, the pro-tumor circuit involves a "Wired" hand-off from tumor cells to astrocytes [54]. This mechanism is a key driver of brain metastasis (BM) colonization, a devastating outcome for patients with breast and lung cancer [54]. This is not a random coupling; brain-metastatic cells *selectively* upregulate Cx43 and, critically, Protocadherin 7 (PCDH7) [54]. PCDH7 functions as a synaptic adapter, forcing the formation of stable, heterotypic Cx43 GJs *specifically* between the cancer cell and the astrocyte [54]. This hijacked "Wired" connection is essential for metastatic survival [62].

The signaling cascade in this pro-tumor circuit mirrors the anti-tumor one, but with a pernicious outcome [62]. cGAMP produced by the metastatic cell's cGAS is transferred via the "Wired" *Cx43/PCDH7* synapse to the astrocyte [62]. This activates STING *in the astrocyte*, causing the astrocyte to secrete IFN-α and TNF-α [54]. These cytokines are *not* anti-tumor in this context [62]. They are released into the metastatic niche and signal in a paracrine fashion *back* to the metastatic tumor cell, activating *its* STAT1 and NF-κB survival pathways [54,62]. This T→Astrocyte paracrine loop provides a continuous stream of pro-survival, pro-growth, and anti-apoptotic signals, rendering the metastatic cells profoundly *resistant* to chemotherapy [54,61,62].

The “Wired vs. Broadcast” framework provides a routing logic for understanding these divergent, recipient-cell-dependent outcomes [38,50]. One level of routing reflects the availability and extent of functional Cx43 GJ coupling [10]. When such coupling is limited (Cx43-low, pS368-high, or low proximity), extracellular “Broadcast” routes may contribute more strongly and remain exposed to ENPP1-mediated hydrolysis [23,47]. When functional Cx43 coupling is available, protected “Wired” transfer may contribute alongside extracellular routes [38,50]. Within the “Wired” component, recipient-cell identity is decisive [54,55]. If the recipient is an APC, the outcome is APC-STING activation, CD8+ T-cell infiltration, and an anti-tumor state [55]. If the recipient is an astrocyte (facilitated by PCDH7), the outcome is astrocyte-STING activation, a paracrine STAT1/NF-κB loop, and a pro-tumor state [54,62]. Cx43 is the conduit; its TME partner dictates its function.

## The broadcast layer: pannexins in extracellular nucleotide signaling

The "Broadcast" layer is the TME's public signaling domain, characterized by contact-independent release and vulnerability to extracellular checkpoints [21]. The primary "Broadcast" channel considered here is Pannexin 1 (PANX1), a large-pore pannexin channel structurally distinct from connexins [63]. While "Wired" Cx43 channels are insulating, PANX1 channels are functionally defined by their *release* of signaling molecules, most notably ATP and UTP, into the extracellular space [21,63]. This ATP "Broadcast" is a critical component of inflammation and immunogenic cell death (ICD) [64].

However, the "Broadcast" of ATP in the TME is rapidly redirected by a highly active ectonucleotidase cascade [24]. This "Broadcast" ATP, a potent pro-inflammatory "find-me" signal, is intercepted by CD39 (ENTPD1) and CD73 (NT5E), which are highly expressed on tumor cells, stromal cells, regulatory T cells (Tregs), and myeloid-derived suppressor cells (MDSCs) [65]. This enzyme axis efficiently converts the "Broadcast" ATP first to AMP (via CD39) and then to immunosuppressive adenosine (via CD73) [24,65]. This locally produced adenosine then activates A2A receptors on T-cells and other immune effectors, *blunting* the anti-tumor immune response and promoting immune evasion [25]. Therefore, the PANX1 "Broadcast" layer, rather than supporting immunity, often *feeds* the immunosuppressive adenosine axis [66,67]. This stands in stark contrast to the protected "Wired" cGAMP signal, which *bypasses* this entire pathway [27,50].

A second "Broadcast" signal is cGAMP itself [50,68]. Tumor cells can export cGAMP into the TME via vesicle-based export, LRRC8 vol-regulated anion channels, and possibly PANX1 channels [68]. Conversely, extracellular cGAMP uptake by recipient cells may involve SLC19A1, LRRC8/VRAC channels, or P2X7-linked mechanisms, depending on cell type and inflammatory context [50,51,68]. This "Broadcast" cGAMP, like "Broadcast" ATP, is highly vulnerable [27]. Its primary adversary is the ectoenzyme ENPP1 (ectonucleotide pyrophosphatase/phosphodiesterase 1), the TME's main extracellular cGAMP hydrolase [27]. High ENPP1 expression, which is common in metastatic tumors like breast cancer, correlates with reduced immune infiltration, resistance to anti-PD-1 therapy, and poor prognosis [27,69].

ENPP1 functions as a dominant innate immune checkpoint by hydrolyzing extracellular “Broadcast” cGAMP before it can activate paracrine STING in host APCs [27]. This creates a high-stakes scenario: in an ENPP1-high TME, extracellular “Broadcast” cGAMP signaling is expected to be strongly attenuated, particularly when importer availability is limited [27,69]. Under these conditions, Cx43-mediated GJIC may provide a protected route for cGAMP transfer that bypasses ENPP1-mediated extracellular loss [27,69].

The "Broadcast" phenotype (pro-tumor, immune-cold) is expected to dominate when "Wired" contacts are low (due to low Cx43 expression or poor T-APC proximity), and/or when "Broadcast" checkpoints are high (high ENPP1, high CD39/CD73) [40]. Under these conditions, neither "Wired" nor "Broadcast" cGAMP efficiently reaches APCs, while "Broadcast" ATP is preferentially converted into adenosine, favoring an immunosuppressive TME [27]. Across several solid tumor settings, ENPP1/CD39/CD73-high states are associated with immune-cold phenotypes and adverse clinical behavior, although their prevalence and effect size likely vary by tumor type and study design [[69], [70], [71]].

## Evidence map and testable translational hypotheses

The "Wired vs. Broadcast" framework is supported by a convergent, although still tumor-context-dependent, body of primary evidence, summarized in Fig. 3. In this map, we distinguish mechanistic/preclinical evidence from clinical or patient-sample correlative evidence to avoid implying that all circuit-level associations have the same level of validation. The anti-tumor T→APC "Wired" circuit (Cx43 → APC → STING) is supported most directly in lung cancer and is also supported by melanoma-related preclinical evidence, where Cx43/cGAMP–STING signaling has been linked to immune-gene signatures, CD8+ T-cell infiltration, and improved response to ICB [40,55]. Conversely, the pro-tumor T→Astrocyte "Wired" circuit (Cx43/PCDH7 → Astrocyte → STING) is established as a mechanism of metastatic fitness and therapy resistance in brain metastasis models from breast and lung cancer [54,62]. Accordingly, the anti-tumor T→APC circuit should currently be interpreted most cautiously as a lung/melanoma-supported module, whereas the pro-tumor T→astrocyte circuit should be interpreted primarily as a brain-metastasis module rather than as a general feature of all tumor sites. Expression of GJA1 and PCDH7 in primary tumors has also been linked to a preferential risk of brain metastasis [54,62].

Taken together, these data support several testable translational hypotheses. First, tumors with a GJA1-high/ENPP1-low state and preserved tumor–APC proximity may be more likely to display APC-STING activation, T-cell-inflamed phenotypes, and sensitivity to anti-PD-1/L1 therapy [40,55,73]. Second, in GJA1+PCDH7+ brain metastases, disruption of the tumor–astrocyte "Wired" circuit warrants evaluation as a strategy to reduce metastatic fitness and chemoresistance [54,62,80,81]. Third, the efficacy of radiotherapy, STING-directed approaches, and Cx43-modulating agents is likely to depend on whether a tumor is wiring-permissive or broadcast-restricted, a distinction that should be tested using spatial and functional biomarkers rather than expression alone [27,68,73,82].

## Therapeutic implications: biomarkers and rational combinations (ICB, radiotherapy, STING agonists)

The “Wired vs. Broadcast” framework provides a context-dependent, hypothesis-generating model for future biomarker and therapeutic-combination studies (summarized in Table 2). Rather than applying uniformly across all cancers, its therapeutic implications should be interpreted according to tumor type, metastatic site, the active "Wired" circuit (T→APC vs. T→Astrocyte), and the dominant "Broadcast" checkpoints (ENPP1, CD39/CD73) [69,70].

**Table 2:** Therapy matrix: Regimen × Prerequisites × Confounders × Translational biomarkers.

Therapeutic Regimen	"Wired/Broadcast" Hypothesis	Prerequisites (Factors to be HIGH)	Confounders (Factors to be LOW)	Translational Biomarkers	Key Evidence
**Anti-PD-1/L1 immune checkpoint blockade (ICB)**	Relies on T→APC "Wired" circuit for baseline T-cell inflammation.	*GJA1* (Cx43), T-APC Proximity, cGAS expression	*ENPP1, CD73, PCDH7*	*GJA1* IHC, Spatial Omics (T-APC contact), ENPP1 expression, CD39/CD73 IHC and enzymatic activity assays	[10.1111/jcmm.70211; 39592436]
**Radiotherapy (RT) + immune checkpoint blockade (ICB)**	Relies on RT-induced cGAMP spreading via "Wired" (Cx43) or "Broadcast" (TME) path.	*GJA1***OR** Low *ENPP1*, cGAS expression	*GJA1*-Low **AND** ENPP1-High ("Double-Locked")	GJA1/ENPP1 IHC, ISG signature post-RT	[10.1186/s13046–021–01826-1]
**"Broadcast" STING Agonist**	Relies on "Broadcast" path (TME) and Import by host cells.	*SLC19A1, STING1* in APCs	*ENPP1* (must be low, or agonist will be degraded)	*ENPP1* expression, *SLC19A1* expression	[10.1073/pnas.2119189119; 35576395]
**RT + ENPP1 Inhibitor**	Aims to *unblock* the "Broadcast" path for RT-induced cGAMP.	cGAS expression, *ENPP1* expression (as a target)	*GJA1* (most relevant in *GJA1*-low tumors)	*ENPP1* IHC, *GJA1* IHC	[10.1038/s43018–020–0028–4 / 33768207]
**GJIC Inhibitor (e.g., Meclofenamate)**	Aims to *break* the pro-tumor T→Astrocyte "Wired" circuit.	*GJA1* (Cx43), *PCDH7*, Astrocyte proximity (Brain Mets)	T-APC Proximity	*GJA1/PCDH*7 IHC in Brain Mets	[10.1038/nature18268; 27225120]

APC, antigen-presenting cell; GJIC, gap junctional intercellular communication; ICB, immune checkpoint blockade; IHC, immunohistochemistry; ISG, interferon-stimulated gene; RT, radiotherapy; TME, tumor microenvironment.

The listed biomarkers and therapeutic scenarios are hypothesis-generating and have not been prospectively validated for patient stratification or treatment selection.

In tumor contexts where it is operative, the T→APC “Wired” circuit may contribute to a T-cell-inflamed TME and immune checkpoint blockade (ICB) responsiveness [40,55,60]. The Cx43 → cGAMP → APC → STING → IFN axis may contribute to baseline CD8+ T-cell recruitment and activation [55,72]. Impaired “Wired” hand-off may therefore reduce the likelihood of ICB responsiveness in these settings [40,69]. A corresponding translational hypothesis is that future biomarker studies should assess features of both “Wired” and “Broadcast” signaling.

Candidate biomarkers for prospective evaluation include GJA1, ENPP1, spatial tumor–APC proximity, and CD39/CD73 activity, because together these variables may inform “Wired” pathway competence and constraints on extracellular cGAMP and ATP signaling [27,40,69,73,74]. High GJA1 is permissive for tumor–APC coupling, whereas high ENPP1 and high CD39/CD73 activity constrain extracellular cGAMP and ATP signaling [27,40,69,74]. Where feasible, phospho-Cx43 markers such as pS368 may further refine this assessment by distinguishing total Cx43 expression from functionally permissive channel states [[8], [9], [10],^39^]. Spatial readouts are essential because GJA1 expression alone cannot establish that GJA1+ tumor cells are actually coupled to STING1+ APCs [73].

This creates a useful working matrix (Fig. 2). A GJA1-high / ENPP1-low state may represent a wiring-permissive phenotype in which both routes remain potentially functional, whereas GJA1-low / ENPP1-high may represent a "Double-Locked" state in which both routes are constrained [27,69,70]. These categories should be viewed as translational models rather than fixed clinical classes and will require prospective validation before they are used for patient selection [27,69,70].

This logic extends directly to radiotherapy (RT) combinations. The immunogenicity of RT, including the bystander and abscopal effects, stems from its induction of dsDNA damage, subsequent cGAS activation, and production of cGAMP [75,76]. This RT-induced cGAMP must *spread* to host APCs to be effective [75,76]. This spread can occur via the "Wired" path (Cx43 GJs are known mediators of the radiation-induced bystander effect) or the "Broadcast" path (RT increases cGAMP export) [75,76].

The "Wired vs. Broadcast" status can therefore inform testable RT-combination hypotheses. In a "Wired-Reliant" tumor (GJA1-high / ENPP1-high), RT-induced cGAMP signaling may rely more heavily on the protected Cx43 route because extracellular "Broadcast" cGAMP is constrained by ENPP1 [27,69]. These tumors may be more likely to benefit from RT + ICB. In a "Broadcast-Reliant" tumor (GJA1-low / ENPP1-low), the "Wired" path is impaired, but the "Broadcast" path remains relatively permissive, allowing RT-induced cGAMP to signal if it survives extracellular hydrolysis and is imported by recipient cells [27,69]. In a "Double-Locked" tumor (GJA1-low / ENPP1-high), RT-induced cGAMP may be produced but may fail to efficiently reach STING-competent APCs because the "Wired" path is impaired and the "Broadcast" path is constrained [27,69]. This phenotype therefore provides a rationale for testing RT + ENPP1 inhibition as a strategy to relieve extracellular cGAMP loss and restore paracrine STING activation [27].

This framework also explains the mixed success of intratumoral STING agonists. These exogenous CDNs (e.g., 2´3´-CDAS) are "Broadcast" signals [77,78]. As such, they are vulnerable to ENPP1 hydrolysis and require an importer, like SLC19A1, to enter recipient cells [77,79]. Their efficacy may therefore be reduced in ENPP1-high or importer-poor tumors [77,79]. The "Wired" transfer of *endogenous* cGAMP via Cx43 remains a mechanistically advantageous delivery mechanism, as it bypasses both extracellular hydrolysis and transmembrane import [27,69].

Finally, the "Wired vs. Broadcast" logic provides a rationale for two distinct classes of channel modulators. The first class, GJIC inhibitors, is designed to *break* pro-tumor "Wired" circuits [80,81]. In the T→Astrocyte circuit, GJs are pro-survival [54,62]. Broad GJIC blockers like the repurposed drugs meclofenamate (MFA) and tonabersat disrupt this circuit [80,81]. Preclinically, they sever the T→Astrocyte paracrine loop, suppress metastatic growth, and restore chemo-sensitivity [80,81]. This strategy is being tested clinically, where MFA is combined with chemotherapy (lomustine, temozolomide) to disrupt the highly "Wired" tumor networks in glioblastoma [80,81].

The second class, scaffolding modulators, aims to *stabilize* or *restore* the anti-tumor T→APC "Wired" circuit. The α-Connexin Carboxyl-Terminal peptide (αCT1) is a cell-penetrating peptide that targets the Cx43 C-terminus [82]. Its mechanism is complex and context-dependent [82]. By disrupting the Cx43–ZO-1 interaction, it can *inhibit* the formation of stable GJ plaques, an effect exploited in canine melanoma models [82]. However, in other models, such as HER2+ breast cancer cells that have lost GJIC, αCT1 can *rescue* communication and enhance sensitivity to targeted therapy [82]. This suggests αCT1 is a true "modulator" that alters the equilibrium between GJs and connexin hemichannels [82]. Its ability to specifically stabilize the T→APC "Wired" synapse without inadvertently promoting connexin hemichannel activity is a critical and unresolved translational question [82].

## Conclusion: key takeaways and translational outlook

The "Wired vs. Broadcast" framework re-contextualizes the role of gap junctions and hemichannels in cancer. It posits that the choice of signaling route, between a private, protected "Wired" transfer via Cx43 GJIC and a public, vulnerable "Broadcast" release via PANX1, is a critical determinant of immune fate. The "Wired" Cx43 GJ functions as a conduit that bypasses the extracellular "Broadcast" checkpoints, namely the cGAMP-hydrolyzing enzyme ENPP1 and the ATP-to-adenosine CD39/CD73 cascade.

This framework provides a compelling mechanistic explanation for one major aspect of the “dual role” of Cx43 in oncology within the cGAMP–STING axis. In this context, Cx43 is not inherently a tumor suppressor or promoter; it behaves as a conduit whose apparent function is dictated by its “Wired” partner and the identity of the recipient cell. When the T→APC "Wired" circuit is engaged, Cx43 is anti-tumor, driving STING-mediated immunity and ICB sensitivity. When the T→Astrocyte "Wired" circuit is hijacked (via PCDH7), Cx43 is pro-tumor, driving brain metastasis and chemoresistance.

Beyond this wiring logic, Cx43 biology also includes additional layers that are not fully captured here, including isoform-specific expression patterns, non-channel signaling functions, and a broader post-translational modification landscape. These dimensions may generate dual or even divergent phenotypes within the same tissue or cell type and should be integrated into future extensions of the framework as more data accumulate.

Several limitations of this review and framework should be acknowledged. First, the "Wired vs. Broadcast" logic has been directly demonstrated only in selected tumor contexts: the anti-tumor T→APC circuit is currently best supported in lung cancer and melanoma-related evidence, whereas the pro-tumor T→astrocyte circuit is most clearly established in brain-metastasis models from breast and lung cancer [40,54,55,62]. Its generalizability to other tumor types, hematological malignancies, non-brain metastatic niches, and non-cGAMP STING agonists remains to be rigorously tested. Second, the "Broadcast" dynamics we describe are intrinsically difficult to quantify in vivo, because short-lived ATP and cGAMP signals and ectoenzyme activities (ENPP1, CD39/CD73) are highly context- and method-dependent. Third, as with any narrative synthesis, we integrate data from heterogeneous experimental models and assays; harmonized spatial-omics and functional GJIC/STING readouts will be required to stress-test and refine this framework in future prospective studies. As such, the “Wired versus Broadcast” model should be viewed as a structured, testable hypothesis rather than a fully validated clinical algorithm.

Taken together, this framework suggests that future translational studies should prospectively evaluate features that inform both “Wired” capacity (e.g., GJA1, PCDH7, and spatial tumor–APC proximity) and “Broadcast” constraints (e.g., ENPP1 and CD39/CD73). In this view, inhibition of pro-tumor–astrocyte coupling, restoration or stabilization of tumor–APC coupling, and pharmacologic relief of extracellular checkpoints represent complementary rather than competing strategies [27,54,62,69,[80], [81], [82]]. Because the TME is a high-interference environment, therapeutic success is likely to depend on whether cGAMP reaches the appropriate recipient cell through a route that is preserved in a given tumor context.

In summary, this routing logic provides testable hypotheses for biomarker development and therapeutic combinations involving ICB, RT, or STING-directed strategies. As harmonized spatial-omics and functional GJIC/STING datasets emerge, these hypotheses can be refined and prospectively evaluated in tumor-type- and site-specific settings.

Box 1 **Methodology and confounders for wired cGAMP hand-off**

Assessing the “Wired” T→APC circuit is methodologically challenging. Although formation of an individual gap junction requires cell–cell contact, tissue-level “Wired” signaling is graded rather than strictly binary because contact frequency, junction abundance, channel permeability, and cGAMP flux vary continuously.

• **Confounder 1: Contact Density & Geometry.** "Wired" transfer is proximity-limited. An "immune-inflamed" tumor with high T-APC contact can use the "Wired" path. An "immune-excluded" or fibrotic tumor cannot, *even if Cx43 expression is high*. This makes spatial biology (e.g., spatial transcriptomics, multiplex IHC) essential for assessing “Wired” potential.
• **Confounder 2: The Cx43 “Phospho-Code.”** Simple GJA1 IHC or mRNA levels are insufficient. A tumor can have high total Cx43 protein that is functionally closed (“unplugged”) due to high PKC-mediated phosphorylation at serine 368 (pS368) or additional phospho-sites such as S279/S282. True “Wired” functional assessment therefore requires a multiplexed approach using phospho-specific antibodies (e.g., anti-pS368, anti-pS279/S282) together with total Cx43 and cell-type markers (for example pan-cytokeratin for tumor cells and CD11c/CD68 for APCs). Techniques such as multiplex immunohistochemistry (mIHC) or imaging mass cytometry (IMC) allow spatially resolved, quantitative assessment of these proteins and their phosphorylation states at cell–cell interfaces, and dynamic functional assays such as dye-transfer or fluorescence recovery after photobleaching (FRAP) can further validate effective gap-junctional coupling.
• **Confounder 3: The "Broadcast" Checkpoints.** The "Wired" path's importance is *relative* to the "Broadcast" path's hostility. A tumor with high ENPP1 expression is expected to rely more heavily on the “Wired” path, because extracellular “Broadcast” cGAMP is more likely to be degraded before reaching STING-competent recipient cells. A tumor with low ENPP1 may successfully use *both* "Wired" and "Broadcast" routes to activate paracrine STING.
• **Confounder 4: GJ Scaffolding & Stability.** The T→Astrocyte "Wire" is selectively stabilized by the adapter PCDH7. The anti-tumor T→APC "Wire" may lack such a specific adapter, making it more transient. The stability of the ZO-1 scaffold and the rate of Cav-1 internalization are key, unmeasured variables.
• **Confounder 5: Measuring “Broadcast” Dynamics and Ectoenzyme Activity.** Quantifying transient ATP and cGAMP concentrations, together with ectoenzyme activity (ENPP1, CD39/CD73), in a dynamic, heterogeneous TME is technically challenging. Signals are rapidly degraded and can vary sharply across micro-regions, and current in situ assays have limited temporal resolution. As suggested by kinetic data for ENPP1 and CD39/CD73, even small increases in ectoenzyme activity can erase most of the Broadcast signal before it reaches APCs, which complicates direct measurement of this layer and demands carefully controlled experimental designs.

A true assessment of the “Wired” circuit requires a systems-level approach: GJA1 (protein level), pS368 (gating status), ZO-1/PCDH7 (scaffolding), T-APC proximity (spatial contact), and ENPP1/CD73 (the “Broadcast” threat).

## Ethics statement

This article is a narrative review based exclusively on previously published literature and does not report any new studies involving human participants, human tissues, or animals performed by the authors. The project was reviewed and approved by the Research Ethics Committees of Qom University of Medical Sciences (Approval ID: IR.MUQ.REC.1405.112; approval date: 19 July 2026). As no new human participants were enrolled and no new human or animal data or specimens were collected, informed consent was not applicable.

## Funding sources

This research did not receive any specific grant from funding agencies in the public, commercial, or not-for-profit sectors.

## Declaration of generative AI and AI-assisted technologies in the manuscript preparation process

During the preparation and revision of this work, the authors used OpenAI’s ChatGPT to assist with literature organization, manuscript structuring, and language and readability refinement. After using this tool, the authors independently verified the sources and scientific content, reviewed and edited the manuscript as needed, and take full responsibility for the content of the published article.

## CRediT authorship contribution statement

**Soroush Akbari-Ardabili:** Writing – original draft, Visualization, Methodology, Conceptualization, Investigation. **Safiyeh Aghazadeh:** Writing – review & editing, Supervision. **Ali Shalizar-Jalali:** Writing – review & editing. **Faezeh Hosseinzadeh:** Writing – review & editing, Resources.

## Declaration of competing interest

The authors declare that they have no known competing financial interests or personal relationships that could have appeared to influence the work reported in this paper.
